# A phase Ib multiple ascending dose study of the safety, tolerability, and central nervous system availability of AZD0530 (saracatinib) in Alzheimer’s disease

**DOI:** 10.1186/s13195-015-0119-0

**Published:** 2015-04-14

**Authors:** Haakon B Nygaard, Allison F Wagner, Garrett S Bowen, Susan P Good, Martha G MacAvoy, Kurt A Strittmatter, Adam C Kaufman, Brian J Rosenberg, Tomoko Sekine-Konno, Pradeep Varma, Kewei Chen, Anthony J Koleske, Eric M Reiman, Stephen M Strittmatter, Christopher H van Dyck

**Affiliations:** Alzheimer’s Disease Research Unit, Yale University School of Medicine, New Haven, Connecticut USA; Department of Neurology, Yale University School of Medicine, New Haven, Connecticut USA; Program in Cellular Neuroscience, Neurodegeneration and Repair (CNNR), Yale University School of Medicine, New Haven, Connecticut USA; Department of Psychiatry, Yale University School of Medicine, New Haven, Connecticut USA; Department of Molecular Biophysics and Biochemistry, Yale University School of Medicine, New Haven, Connecticut USA; Department of Diagnostic Radiology, Yale University School of Medicine, New Haven, Connecticut USA; Banner Alzheimer’s Institute, Phoenix, Arizona USA; Current address: University of British Columbia, Division of Neurology, Djavad Mowafaghian Centre for Brain Health, Vancouver, Canada

## Abstract

**Introduction:**

Despite significant progress, a disease-modifying therapy for Alzheimer’s disease (AD) has not yet been developed. Recent findings implicate soluble oligomeric amyloid beta as the most relevant protein conformation in AD pathogenesis. We recently described a signaling cascade whereby oligomeric amyloid beta binds to cellular prion protein on the neuronal cell surface, activating intracellular Fyn kinase to mediate synaptotoxicity. Fyn kinase has been implicated in AD pathophysiology both in *in vitro* models and in human subjects, and is a promising new therapeutic target for AD. Herein, we present a Phase Ib trial of the repurposed investigational drug AZD0530, a Src family kinase inhibitor specific for Fyn and Src kinase, for the treatment of patients with mild-to-moderate AD.

**Methods:**

The study was a 4-week Phase Ib multiple ascending dose, randomized, double-blind, placebo-controlled trial of AZD0530 in AD patients with Mini-Mental State Examination (MMSE) scores ranging from 16 to 26. A total of 24 subjects were recruited in three sequential groups, with each randomized to receive oral AZD0530 at doses of 50 mg, 100 mg, 125 mg, or placebo daily for 4 weeks. The drug:placebo ratio was 3:1. Primary endpoints were safety, tolerability, and cerebrospinal fluid (CSF) penetration of AZD0530. Secondary endpoints included changes in clinical efficacy measures (Alzheimer’s Disease Assessment Scale – cognitive subscale, MMSE, Alzheimer’s Disease Cooperative Study – Activities of Daily Living Inventory, Neuropsychiatric Inventory, and Clinical Dementia Rating Scale – Sum of Boxes) and regional cerebral glucose metabolism measured by fluorodeoxyglucose positron emission tomography.

**Results:**

AZD0530 was generally safe and well tolerated across doses. One subject receiving 125 mg of AZD0530 was discontinued from the study due to the development of congestive heart failure and atypical pneumonia, which were considered possibly related to the study drug. Plasma/CSF ratio of AZD0530 was 0.4. The 100 mg and 125 mg doses achieved CSF drug levels corresponding to brain levels that rescued memory deficits in transgenic mouse models. One-month treatment with AZD0530 had no significant effect on clinical efficacy measures or regional cerebral glucose metabolism.

**Conclusions:**

AZD0530 is reasonably safe and well tolerated in patients with mild-to-moderate AD, achieving substantial central nervous system penetration with oral dosing at 100–125 mg. Targeting Fyn kinase may be a promising therapeutic approach in AD, and a larger Phase IIa clinical trial of AZD0530 for the treatment of patients with AD has recently launched.

**Trial registration:**

ClinicalTrials.gov: NCT01864655. Registered 12 June 2014.

## Introduction

Despite considerable ongoing efforts to halt or reverse the symptoms of Alzheimer’s disease (AD), a disease-modifying intervention for this devastating illness has not yet emerged. The major approach to AD therapeutic development has been to target amyloid-beta (Aβ), by either limiting its cleavage from the amyloid precursor protein, or facilitating its clearance by active or passive immunization [1]. An alternative approach to AD treatment is to target the downstream effects of pathologic Aβ signaling, without altering protein levels. This may be a particularly attractive approach as some forms of Aβ could have important physiologic roles [2,3]. More recently, preclinical focus has changed from insoluble assemblies of Aβ to oligomeric species (Aβ_o_) and, while the precise nature of the toxic Aβ_o_ is not fully understood, a unifying theme among various preparations and sources is synaptotoxicity [4-6]. Blocking this detrimental downstream effect of Aβ_o_ may be a key feature of future Aβ-based therapeutics in AD.

The mechanism by which Aβ_o_ causes synaptoxicity is of significant interest, with a leading hypothesis postulating the existence of a distinct cell surface receptor mediating its effects. To identify such a potential binding site of Aβ_o_ on neurons, we performed a genome-wide unbiased screen for cell surface proteins binding Aβ_o_, identifying cellular prion protein (PrP^C^) as a high-affinity receptor [5]. Aβ binding to PrP^C^ is highly conformation-dependent, and only Aβ_o_ will interact with PrP^C^, with no binding detected in the presence of fibrillary or monomeric Aβ peptide [5,7-9]. Emphasizing its potential importance, PrP^C^ is required for most deficits seen in amyloid precursor protein/presenilin 1 transgenic mice, including spatial learning and memory deficits, reduced survival, epileptiform discharges, synapse loss, serotonin fiber degeneration and for cell death *in vitro* [10-17]. Human AD brain-derived Aβ species suppress hippocampal synaptic plasticity *in vivo* in a PrP^C^-dependent fashion, and human AD brain extracts contain Aβ_o_ species that interact with PrP^C^ as well as Aβ-PrP^C^ complexes [10,18-20]. Several groups, including ours, recently showed that the Aβ_o_-PrP^C^ complex activates an intracellular signaling cascade coupled to the protein tyrosine kinase Fyn [5,10,21,22]. This is of particular interest since Fyn has been implicated in AD pathogenesis across various models, including human subjects [23]. Fyn is central to Aβ signal transduction, and also has major functional interactions with Tau [24-27], thereby unifying the two principal pathologies in AD. Thus, blocking Fyn kinase may be a promising therapeutic approach in AD.

Saracatinib (AZD0530) is a small molecule inhibitor of the Src family kinases, blocking Src, Fyn, Yes and Lyn, with 2 to 10 nM potency [28]. At 10- to 100-fold higher concentrations the compound also inhibits Abl family kinases, without detectable activity in this concentration range against other kinase families, including c-kit, Csk and platelet-derived growth factor. Due to its specific inhibition of Fyn and Src family kinases, and the fact that Src family kinases regulate cell proliferation and tumor cell adhesion, migration and invasion, AZD0530 was initially developed as a therapy for solid tumors [28]. To date, more than 500 subjects have received once-daily oral doses of AZD0530. Preliminary studies indicate that the dose needed to disrupt the Aβ-PrP^C^-Fyn signaling cascade is significantly lower than that needed for cancer therapy, and doses up to 125 mg daily have been found to be safe and well tolerated (unpublished data).

Herein we present the results of a 4-week multiple ascending dose study of the safety, tolerability, and central nervous system (CNS) availability of AZD0530 (saracatinib) in mild-to-moderate AD. We assessed the safety and tolerability of three escalating doses of AZD0530, as well as the ability of the compound to penetrate the CNS, a critical feature for any AD drug. Secondary aims included changes in clinical efficacy measures and in regional cerebral glucose metabolism.

## Methods

### Study protocol

This study protocol was reviewed and approved by the Yale Human Investigation Committee. All study participants and their study partners provided written informed consent to participate in this study. Given the absence of potential benefit in this short Phase Ib study, participants were required to provide informed consent for themselves (that is, surrogate consent was not permitted).

### Study population

Men and women aged 50 to 90 years old (inclusive) with a diagnosis of probable AD dementia based on the National Institute on Aging-Alzheimer’s Association core clinical criteria [29], and Mini-Mental State Examination (MMSE) [30] score of 16 to 26 were considered for study participation. Additionally, subjects were required to have stable permitted medications for 4 weeks prior to study participation. Cholinesterase inhibitors and memantine were required to be stable for 12 weeks prior to screen. Participants also needed to score less than 6 on the Geriatric Depression Scale [31] and less than or equal to 4 on the Modified Hachinski Scale [32]. Additionally, they needed to have completed six grades of education or have a good work history and speak English fluently. Amyloid positron emission tomography (PET) imaging was not used to determine study eligibility.

Participants were excluded from the study if they exhibited any significant neurologic disease other than AD. Additionally, subjects with a screening magnetic resonance imaging scan showing evidence of infection, infarction, or other focal lesions as well as multiple lacunes or lacunes in a brain region critical for memory were excluded. A history of schizophrenia or of alcohol or substance abuse/dependence within the past 2 years (Diagnostic and Statistical Manual of Mental Disorders, 4th edition criteria) was also exclusionary. Any subject with a clinically significant or unstable medical condition that could have put the subject at risk due to study participation, or influence the results, was excluded. Additionally, the following laboratory results were considered exclusionary: clinically significant abnormalities in vitamin B12 or thyroid function tests that might interfere with the study, neutropenia defined as an absolute neutrophil count of <1,500/μl, thrombocytopenia defined as platelet count <100 × 10^3^/μl, current blood clotting or bleeding disorder, or significantly abnormal prothrombin time or partial thromboplastin time at screening, or clinically significant abnormalities in other screening laboratories, including aspartate aminotransferase >1.5 × upper limit of normal (ULN); alanine aminotransferase >1.5 × ULN; total bilirubin >1.5 × ULN; serum creatinine >2.0 × ULN. Additionally, a history of interstitial lung disease was exclusionary. Residence in a skilled nursing facility was also considered exclusionary.

Current use (within 30 days of screening) of the following medications was exclusionary: typical neuroleptics, narcotic analgesics, Parkinson’s medications, systemic corticosteroids, or medications with significant central anticholinergic activity, or warfarin. Current use (within 30 days of screening and throughout the protocol including the 2-week follow-up period) of the following medications was exclusionary: a) strong CYP3A4 inhibitors including: atazanavir, indinavir, ritonavir, saquinavir, nelfinavir, ketoconazole, itraconazole, clarithromycin, telithromycin, and nefazodone; b) strong CYP3A4 inducers including: rifampicin, phenytoin, phenobarbital, and carbamazepine; c) certain CYP3A4 substrates including colchicine, cyclosporine, disopyramide, fluticasone, quinidine, vinblastine, vincristine. Subjects taking sildenafil, tadalafil, and vardenafil were advised to stop taking these medications for the duration of the trial.

### Study design

The study was conducted in the Yale Alzheimer’s Disease Research Unit between July 2013 and March 2014. This 4-week, multiple ascending dose, randomized, double-blind, placebo-controlled, Phase Ib trial enrolled 24 subjects. The subjects were divided into three sequential cohorts. Each cohort contained six subjects assigned to active drug and two subjects assigned to placebo. The dose of the first cohort was 50 mg of oral AZD0530 or placebo per day for 4 to 5 weeks. The dose of the second and third cohorts was to be determined from the results of cohort 1 and ongoing preclinical studies, not to exceed 125 mg daily. Each cohort of eight participants completed the study before the next cohort was recruited.

The primary aims of this study were to assess the safety and tolerability of oral AZD0530 in patients with AD and to determine dose levels that are well tolerated and provide cerebrospinal fluid (CSF) concentrations predicted to slow AD progression. Secondary aims were to assess effects of AZD0530 on clinical efficacy assessments and brain ^18^F-fluorodeoxyglucose (FDG) PET changes in a statistical region of interest (sROI) known to be preferentially affected in AD over longer time periods.

Potential participants who were found to be eligible based on the initial screening evaluation proceeded to the baseline assessments. These included safety assessments, clinical efficacy assessments, ^18^F-FDG PET imaging, collection of AZD0530 metabolites and AD biomarkers, and dispensation of study medication. Subjects were seen for safety visits at weeks 1, 2, and 3. Outcome measures were collected after 4 weeks on AZD0530 or placebo, including measures of safety and tolerability, CSF drug concentration, clinical efficacy measures, and ^18^F-FDG PET imaging. Study medication could be continued for up to 5 weeks (35 days) in order to complete all outcome measures. Study medication compliance was measured throughout the study. A final safety visit was completed after the participant had been off AZD0530 or placebo for approximately 2 weeks.

### Safety assessments

Safety assessments included physical and neurological examinations, MMSE examinations, vital signs (including blood pressure, pulse, oral temperature, respiration rate, and weight), electrocardiograms, and clinical laboratories. Physical and neurological examinations occurred at all visits except the baseline visit. Electrocardiograms occurred at the screening, and at week 4 and week 6 visits. Vital signs, clinical laboratories, and MMSE examinations occurred at all visits. Previous clinical experience with AZD0530 has indicated a possible, but rare, relationship with interstitial lung disease in patients with advanced solid tumors [33]. For this reason, thoracic high-resolution computed tomography was obtained if unexplained pulmonary symptoms arose at any point during the study.

### Clinical efficacy measures

At the baseline and week 4 visits, subjects underwent the following clinical efficacy assessments: Alzheimer’s Disease Assessment Scale – cognitive subscale (ADAS-cog) [34], MMSE [30], Alzheimer’s Disease Cooperative Study – Activities of Daily Living Inventory (ADCS-ADL) [35], Neuropsychiatric Inventory (NPI) [36], and Clinical Dementia Rating Scale – Sum of Boxes (CDR-SOB) [37]. The MMSE was also completed at screening and weeks 1, 2, and 3.

### ^18^F-FDG PET imaging

Eligible subjects had ^18^F-FDG PET brain scans performed at the baseline visit and at week 4. A 30-minute dynamic emission scan, consisting of six 5-minute frames was acquired starting 30 minutes after the intravenous injection of 5 mCi of ^18^F-FDG. Data was corrected for radiation-attenuation and scatter using transmission scans or X-ray computed tomography, and reconstructed using a standardized reconstruction algorithm.

### Peripheral Src family kinase target engagement

Osteoclast function is known to be mediated through Src, and therefore levels of cross-linked serum C-telopeptide of type 1 collagen (sCTX) measurements have been used in several studies as a surrogate marker of relevant biologic function of AZD0530 [38,39]. sCTX levels were measured at the baseline visit and at week 4 by enzyme-linked immunosorbent assay (Immunodiagnostic Systems, Boldon, UK) according to the manufacturer’s instructions. Serum samples were stored at −80°C until analysis. All samples collected from each individual subject were analyzed in the same batch by an investigator blinded to subject drug assignment.

### Abl inhibition assay

An assay for plasma inhibitory activity against Abl kinase followed previous procedures [40,41] by determining the level of Abl-dependent phosphorylation of Stat5 in leukemia cells incubated with plasma from patients. The K-562 cells, a gift from Prof. Gary Kupfer, were maintained in RPMI 1640 supplemented with 10% fetal bovine serum. To generate a dose–response curve for AZD0530 and STI-571, 1 × 10^5^ K-562 cells were incubated in 100 μl normal volunteer plasma in 96 well plates for 2 hours at 37°C in a humidified incubator with 5% CO_2_, with the appropriate amount of drug added, diluted in equal volumes of DMSO. For experiments with patient samples, cells were incubated in patient plasma samples, with the experimenter blinded to patient group. Cells were pelleted by centrifuge, washed twice in 500 μl phosphate-buffered saline, then lysed in 40 μl 2× Laemmli sample buffer (LSB), boiled for 10 minutes and centrifuged. Lysates were then loaded onto 8% SDS-PAGE gels, transferred to nitrocellulose membranes, and immunoblotted using a phospho-specific Stat5 antibody (Cell Signaling, Danvers, MA, USA), and then stripped and re-probed using a Stat5 antibody (SantaCruz, Dallas, TX, USA). Immunoblots were then quantified by densitometry, and the density of each band normalized to a control sample treated with volunteer plasma. Nonlinear regression was performed to generate a dose–response curve of inhibition versus AZD0530 and STI concentration.

### Cerebrospinal fluid Alzheimer’s disease biomarkers

Levels of CSF Aβ40, Aβ42, Tau and P231-Tau were measured using a Luminex assay system and a MagPix instrument, according to the manufacturer’s instructions (Millipore, Billerica, MA, USA). All assays were performed in a single batch by an investigator blinded to subject assignments.

### Rodent AZD0530 pharmacokinetics

To examine the correlation between plasma, CSF, and brain levels of AZD0530 in a preclinical model, five mice (C57B6/J) received a dose of 5 mg/kg per day administered orally twice daily for 3 days. Trough plasma, CSF and brain drug levels were measured by tandem reverse-phase high-pressure liquid chromatography and mass spectrometry. All animal work was approved by the Yale Institutional Animal Care and Use Committee according to established international guidelines.

### Statistical analysis

At the completion of all three dose cohorts, the frequencies of adverse events or laboratory abnormalities between the participants who received each dose of AZD0530 (n = 6) and the pooled placebo subjects (n = 6) were compared using Fisher’s exact test. Changes in clinical outcomes (MMSE, ADAS-cog, CDR-SOB, ADCS-ADL, NPI) were examined using analysis of covariance or Kruskal-Wallis H test (for non-normally distributed NPI data). Change in ^18^F-FDG PET cerebral metabolic rate for glucose (CMRgl) from baseline to week 4 was analyzed using statistical parametric mapping sROI methods as previously published [42]. The effect of treatment assignment on change in CMRgl was analyzed by four-group analysis of variance and *t*-test.

## Results

### Subject disposition

Thirty-one subjects were screened, with 24 meeting eligibility criteria. Seven subjects were excluded as follows: inability to consent (2), MMSE >26 (2), abnormal thyroid stimulating hormone (1), thalamic infarct (1), and abnormal liver function tests (1). Of the 24 subjects, 18/24 were randomized to one of three doses of AZD0350, and the remaining six to placebo. One subject discontinued treatment prematurely due to a serious adverse event, as detailed below.

### Baseline characteristics

Of the 24 subjects, 61% were female, with age ranging from 55 to 86 years (mean ± SD, 73 ± 7) (Table 1); 96% of subjects were Caucasian. Mean score on the MMSE at baseline was 22.2 (±3). The treatment groups did not differ significantly on any demographic or baseline clinical efficacy variable. However, the 125-mg group was slightly older and had a lower baseline MMSE score in comparison to other groups.

**Table 1:** **Subject characteristics and clinical efficacy assessments**

**Variable**	**Treatment group (dose of AZD0530)**				
	**Placebo (n = 6)**	**50 mg (n = 6)**	**100 mg (n = 6)**	**125 mg (n = 6)**	***F P***
Age	72.6 ± 9.5	72.6 ± 4.4	71.5 ± 4.9	4.6 ± 7.9	0.57 † 0.19
Sex	3 F, 3 M	5 F, 1 M	2 F, 4 M	3 F, 3 M	
Education (years)	15.0 ± 2.8	15.3 ± 2.7	14.3 ± 2.3	16.8 ± 3.4	0.84 † 0.49
Weight (kg)	6.88 ± 12.1	69.7 ± 8.7	79.6 ± 17.9	65.4 ± 10.4	1.37 † 0.28
MMSE, baseline	23.0 ± 4.1	21.8 ± 3.3	22.2 ± 4.6	20.2 ± 2.3	0.63 † 0.60
MMSE, week 4	25.7 ± 3.3	25.3 ± 2.4	22.7 ± 4.0	22.5 ± 3.3	
MMSE, change	2.7 ± 2.0	3.5 ± 1.9	0.5 ± 3.4	2.3 ± 2.1	2.15 § 0.13
ADAS-cog, baseline	16.7 ± 2.3	20.6 ± 6.7	23.8 ± 4.5	21.6 ± 7.1	1.73 † 0.19
ADAS-cog, week 4	15.2 ± 4.8	18.3 ± 4.5	22.3 ± 8.1	23.0 ± 4.4	
ADAS-cog, change	−1.6 ± 4.4	−2.3 ± 3.4	−1.4 ± 5.4	1.4 ± 3.2	1.31 § 0.30
ADCS- ADL, baseline	63.5 ± 9.2	68.7 ± 6.8	67.2 ± 6.4	64.8 ± 3.1	0.67 † 0.58
ADCS- ADL, week 4	67.7 ± 10.6	66.5 ± 6.7	68.2 ± 7.6	66.4 ± 4.3	
ADCS- ADL, change	4.2 ± 5.2	−2.2 ± 4.2	1.0 ± 2.0	1.6 ± 3.4	2.27 ‡ 0.12
CDR-SOB, baseline	5.6 ± 2.2	5.3 ± 1.9	4.8 ± 1.2	5.1 ± 1.8	0.22 † 0.88
CDR-SOB, week 4	5.8 ± 2.4	5.0 ± 1.9	4.8 ± 1.2	5.6 ± 1.9	
CDR-SOB, change	0.2 ± 0.4	−0.3 ± 0.6	0.0 ± 0.0	0.5 ± 0.4	2.60 ‡ 0.08
NPI, baseline	9.5 ± 11.9	6.5 ± 6.1	3.7 ± 4.3	13.2 ± 10.1	¶ 0.37
NPI, week 4	4.0 ± 5.0	7.7 ± 9.1	2.5 ± 3.5	18.0 ± 8.9	
NPI, change	−5.5 ± 8.4	1.2 ± 5.1	−1.2 ± 4.8	4.8 ± 7.6	¶ 0.09

Values are shown as mean ± standard deviation (apart from sex). *F* and *P* values are for analysis of covariance, controlling for baseline score. ^†^Analysis of variance. ^¶^Kruskal-Wallis H test. ^‡^Analysis of completer subjects only. Week 4 ADCS-ADL and CDR-SOB invalid for subject AZD026, who was residing in skilled nursing facility. ^§^Analysis of all randomized subjects, including AZD026 who discontinued study drug after 9 days; excluding this subject, MMSE change in 125-mg group = 2.8 ± 1.9, *F* = 2.07, *P* = 0.140; ADAS-cog change = 1.3 ± 3.5, *F* = 1.05, *P* = 0.393; NPI change = 1.8 ± 1.9, *P* = 0.169, Kruskal-Wallis H test. ADAS-cog, Alzheimer’s Disease Assessment Scale – cognitive subscale; ADCS-ADL, Alzheimer’s Disease Cooperative Study – Activities of Daily Living Inventory; CDR-SOB, Clinical Dementia Rating Scale – Sum of Boxes; F, female; M, male; MMSE, Mini Mental Status Examination; NPI, Neuropsychiatric Inventory.

### Safety and tolerability

In general, doses of AZD0530 ranging from 50 mg to 125 mg daily were reasonably well tolerated in patients with mild-to-moderate AD. The number of participants in each AZD0530 treatment group experiencing adverse events is presented in Table 2. Treatment-emergent adverse events were reported for three participants in the placebo group, and four, five, and four participants in the 50-mg, 100-mg, and 125-mg groups, respectively. All adverse events were of mild or moderate severity. Two participants in the 125-mg dose group had moderately severe events, compared to one each for the 100-mg and placebo groups. The most common individual adverse events were diarrhea, headache, fatigue, and nausea, which tended to occur more frequently with higher doses of AZD0530 but were numerically most frequent in the placebo group. The effect of treatment group was not statistically significant for any adverse event.

**Table 2:** **Number of participants in each AZD0530 treatment group experiencing an adverse event**

	**Treatment group (AZD0539 dose; n = 6 per group)**				
**Adverse event**	**Placebo**	**50 mg**	**100 mg**	**125 mg**	**Total**
Any adverse event*	**3**	**4**	**5**	**4**	16
Diarrhea	2	0	1	2	5
Headache	2	1	1	1	5
Fatigue	1	0	0	2	3
Nausea	2	0	0	1	3
Pneumonia, atypical/bronchitis	0	0	0	1	1
Congestive heart failure	0	0	0	1	1
Renal insufficiency, worsened	0	0	0	1	1
Elevated serum creatinine	0	0	0	1	1
Cough, worsened	0	0	0	1	1
Postnasal drip	0	0	0	1	1
Pulmonary hypertension	0	0	0	1	1
Anorexia	0	0	0	1	1
Tinnitus	0	0	0	1	1
Myalgias	0	0	0	1	1
Squamous cell carcinoma	0	0	1	0	1
Basal cell carcinoma	0	0	1	0	1
Wrist pain	0	0	1	0	1
Noncardiac chest pain	0	0	1	0	1
Upper respiratory infection	0	1	0	0	1
Flu-like symptoms	0	1	0	0	1
Lightheadedness	0	1	0	0	1
Vomiting	1	0	0	0	1

*Includes participants who reported at least one adverse event. The effect of treatment group is not significant for any adverse event or individual adverse events (Fisher’s exact test, *P* > 0.05).

The only serious adverse event (SAE) occurred in a participant who was receiving 125 mg AZD0530. After 9 days of treatment, the subject was hospitalized with 4 days of fatigue, anorexia, and myalgias, and 1 day of shortness of breath. A low-grade fever with a maximum temperature of 100.1 **°**F was recorded after hospital admission. The diagnosis was felt to be most consistent with congestive heart failure, precipitated by pneumonia; however, a drug-induced pneumonitis could not be entirely ruled out. An infectious organism was not isolated. Study medication was discontinued, and the subject was treated with diuretics and antibiotics, gradually making a full recovery to baseline health. The overall conclusion was that this event was possibly related to the study drug. Apart from this SAE, no other participant prematurely discontinued study medication.

Two subjects receiving AZD0530 125 mg daily, one of which experienced the SAE described, had significant laboratory abnormalities that subsequently reversed: elevated serum creatinine, considered possibly related to study medication, and worsened renal insufficiency, unlikely related to study medication. There were no significant hematological changes, including platelet counts and absolute neutrophil counts. No significant changes in measures of vital signs or electrocardiogram readings were seen across AZD0530 doses.

### AZD0530 drug levels in human cerebrospinal fluid at different doses

**Figure 1:**
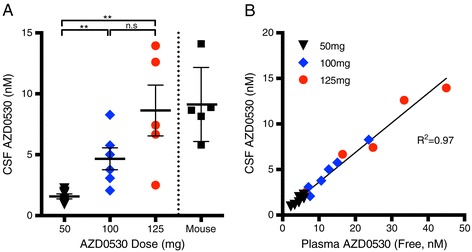
**AZD0530 in human cerebrospinal fluid at different doses. (A)** Each point represents fasting trough cerebrospinal fluid (CSF) AZD0530 level from a different human subject for the left three columns. The mouse trough CSF values are derived from brain levels at the 5 mg/kg per day dose that rescued memory deficits [43]. ** *P* < 0.001, one-way analysis of variance with Tukey *post-hoc* comparisons. **(B)** Relationship between CSF and plasma AZD0530 levels. Each point is from a different individual, and different dose groups are illustrated with different colors. There is a tight correlation of plasma and CSF level as indicated (R^2^ = 0.97, Pearson correlation coefficient). Bars represent mean ± standard error of the mean. n.s, Not significant.

We compared the CSF drug levels across the AZD0530 dosage groups. There was variability within any one group, but dose dependency was established (Figure 1A). The increase in CSF drug level was greater than linear, with a three-fold increase from 50 to 100 mg and a two-fold increase from 100 to 125 mg, most consistent with an exponential relationship. The trough CSF drug level at 50 mg was 0.5 to 1.2 ng/ml (0.9 to 2.2 nM). The trough CSF drug level at 100 mg was 1.1 to 4.5 ng/ml (2.1 to 8.3 nM). The trough CSF drug level at 125 mg was 1.4 to 7.6 ng/ml (2.5 to 14.0 nM). Using the ratio of CSF:brain in mice measured to be 1:3, the estimated human brain concentrations at 50 mg = 3 to 7 nM, at 100 mg = 7 to 27 nM and at 125 mg = 8 to 46 nM. The Fyn Ki for AZD0530 is 5 to 10 nM. Analysis of the preclinical efficacy of AZD0530 in a mouse model of AD is reported elsewhere [43]; 5 mg/kg per day administered for 4 to 6 weeks reverses memory impairments in this model, corresponding to trough CSF levels in humans seen at doses of 100 to 125 mg/day (Figure 1A).

We collected both CSF and plasma for AZD0530 drug levels at the week-4 endpoint. There was a close correlation between free AZD0530 drug levels in plasma and CSF AZD0530 (Figure 1B). CSF drug levels are about one-third of plasma free drug levels (Figure 1B).

### Clinical efficacy measures

All subjects underwent standard clinical efficacy assessments at baseline and at the week-4 endpoint (Table 1). There was a tendency for MMSE scores to improve over time, which may have been due to practice effects as the MMSE was also administered at weekly visits as a safety measure. No statistically significant effect of treatment was observed on any of clinical efficacy assessment measures, including ADAS-cog, ADCS-ADL, CDR-SOB, and NPI. The lack of a treatment effect was not surprising given the small sample sizes and the short duration of treatment, and with an investigational therapy that is being developed primarily for longer-term disease- modifying potential.

### ^18^F-FDG PET measurements

The effect of treatment with AZD0530 on 1-month reductions in CMRgl using statistical parametric mapping sROI was assessed using ^18^F-FDG PET imaging. Of the 24 subjects, 22 underwent measurements of CMRgl at baseline, and after 4 weeks on study drug or placebo. There was no effect of treatment group on change in a prespecified sROI consisting of voxels associated with preferential 12-month CMRgl declines in previously studied patients with the clinical diagnosis of mild-to-moderate AD dementia (*F* = 0.37, *P* = 0.78, df = 3.17, analysis of variance). A *post-hoc* comparison of change in CMRgl for clinically relevant doses of AZD0530 (100 or 125 mg daily) versus placebo revealed no significant differences in the rate of decline for CMRgl between AZD0530 100 to 125 mg daily (0.87 ± 1.86%) versus placebo (1.26 ± 2.24%; *P* = 0.71).

### Peripheral target engagement

**Figure 2:**
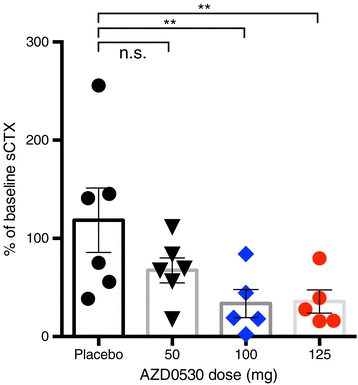
**AZD0530 peripheral target engagement.** Four-week treatment with 100 mg and 125 mg AZD0530 reduces serum cross-linked C-telopeptide of type 1 collagen (sCTX), a marker of osteoclast activity. Compared to placebo, mean difference in the change in sCTX from baseline for 50 mg was 51% (95% confidence interval, −22.0 to 124.3), for 100 mg 85% (95% confidence interval, 8.2 to −161.6), and for 125 mg 83% (95% confidence interval, 6.2 to 159.6). **P < 0.05, analysis of variance with Dunnet’s multiple comparisons test. n.s., Not significant.

To provide a readily accessible marker of peripheral Src family kinase target inhibition, we assayed sCTX. This collagen fragment is a marker of activity for the osteoclast, a cell in which Src plays an important regulatory role. AZD0530 is reported to decrease sCTX in humans in a dose-dependent fashion [38]. Our results confirm Src peripheral target engagement, with a decrease in serum sCTX after 4 weeks of daily 100- to 125-mg AZD0530 administration (Figure 2).

### AZD0530 does not inhibit Abl kinase at doses up to 125 mg

**Figure 3:**
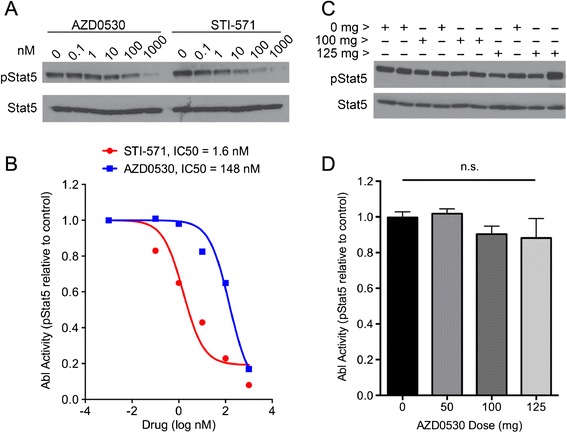
**Plasma from AZD0530-treated subjects does not inhibit Abl signaling to Stat5. (A)** The level of pStat5 and total Stat5 in K-562 cells incubated with the indicated concentrations of AZD0530 or STI-571 is shown. **(B)** The pStat5 level from experiments in (A) was measured by microdensitometry and a half maximal inhibitory concentration (IC50) determined for each compound. **(C)** The plasma inhibitory activity for Abl was detected by pStat5 level in K-562 cells incubated with plasma from subjects treated with the indicated doses of AZD0530. **(D)** The plasma Abl inhibitory activity determined by pStat5 level was measured in all subjects, and is plotted as mean ± standard error of the mean. No significant difference between groups was observed (n.s.).

AZD0530 has highest activity against Src family kinases, with little or no potency against a panel of other kinases. The only other detectable activity is against Abl kinase, with a Ki approximately 15-fold higher [28,44]. Therefore, we measured the activity of plasma inhibitory activity of AZD0530 against Abl kinase, by monitoring the level of Stat5 phosphorylation as a substrate of Abl in leukemia cells as described [40,41]. While the Abl kinase inhibitor STI-571 inhibits Abl to Stat5 signaling with a half maximal inhibitory concentration (IC50) of 1.6 nM in cells incubated with plasma, the IC50 for AZD0530 is nearly 100-fold higher, at 156 nM (Figure 3A,B). Plasma samples from subjects treated with 0, 50, 100 and 125 mg were incubated with the leukemia cells *in vitro*, and pStat5 measured as a read out of Abl kinase activity (Figure 3C,D). No significant inhibition of Abl kinase was observed. Thus, at these doses of AZD0530, kinase inhibition is specific for the Src family (Figure 2), without significant alterations in Abl kinase activity (Figure 3D).

### Cerebrospinal fluid biomarkers

Levels of total Aβ40, Aβ42, Tau, and p-Tau were assessed in CSF samples from all subjects in the study after 4 weeks of study medication. Although we did not obtain CSF at baseline to analyze drug effects on CSF biomarkers, we found no difference in levels of any AD biomarker at the 4-week endpoint between subjects treated with 100 to 125 mg AZD0530 and placebo (for Aβ40: placebo, 4,876 pg/ml (±789 pg/ml); AZD0530 100 mg, 3,963 pg/ml (±624 pg/ml); AZD0530 125 mg, 4,501 pg/ml (±311 pg/ml); for Aβ42: placebo, 601 pg/ml (±81 pg/ml); AZD0530 100 mg, 523 pg/ml (±48 pg/ml); AZD0530 125 mg, 553 pg/ml (±34 pg/ml); for total tau: placebo, 0.53 ng/ml (±0.17 ng/ml); AZD0530 100 mg, 0.57 ng/ml (±0.20 ng/ml); AZD0530 125 mg, 0.57 ng/ml (±0.14 ng/ml); for p(T231)-Tau: placebo, 5.33 nM (±2.29 nM); AZD0530 100 mg, 5.88 nM (±1.89 nM); AZD0530 125 mg, 5.21 nM (±1.80 nM)).

## Discussion

Preclinical studies in both rodent models and patients with AD indicate that Fyn may be a promising target for novel therapeutic intervention [23]. Here, we report a Phase Ib study to explore the use of a Fyn-specific Src family kinase inhibitor for the treatment of patients with AD. Our study shows that 100 mg and 125 mg doses achieved CSF levels corresponding to brain levels that rescued memory deficits in transgenic AD mouse models. A therapeutic benefit was not expected after a 1-month treatment period, and no treatment effect was observed across doses of AZD0530 on measures of cognitive and neuropsychiatric function, activities of daily living, or cerebral glucose metabolism.

Some safety and tolerability issues may emerge, particularly at the 125 mg dose. However, the tight correlation between plasma and CSF levels of AZD0530 will enable individualized dosing within the 100 to 125 mg range, based on early plasma level monitoring. Individualized dosing may maximize the number of subjects who reach the target CSF drug concentration of 5 nM, while minimizing those who encounter safety and tolerability problems. Fyn regulates a diverse set of cellular functions, including cell proliferation, migration, and differentiation, synaptic function, CNS myelination, T cell signaling, and platelet function [23], emphasizing not only the importance of careful safety and tolerability monitoring in larger clinical trials, but also the goal of normalizing aberrant Fyn activity in AD, without significantly altering its physiologic functions. Previous experience with AZD0530 has indicated a possible, but rare, relationship with interstitial lung disease in patients with advanced solid tumors [33]. The single SAE in this study involved a case of congestive heart failure and pneumonia, for which a drug-induced pneumonitis could not be entirely ruled out. Although no conclusive link has been established between AZD0530 and drug-induced pneumonitis, future trials of AZD0530 for AD should continue to monitor this possibility with thoracic high-resolution computed tomography for any unexplained pulmonary symptoms. We recognize the limitations of our results due to the relatively small sample size and the short duration of treatment. These limitations will be addressed by a larger Phase IIa clinical trial of AZD0530 in AD (NCT02167256).

Masitinib, a tyrosine kinase inhibitor selective for c-Kit, platelet-derived growth factor, Lyn, and to a lesser degree Fyn, was recently used in a 24-week, Phase II dose-ranging trial in France, involving 34 patients with mild-to-moderate AD [45]. The trial showed reasonable tolerability, and drug treatment was associated with improvements in cognition and daily function at 12 and 24 weeks. A large international Phase III trial was launched in 2013 to evaluate the efficacy and safety of two doses of masitinib compared to placebo (NCT01872598). The Phase II data provide further clinical support for tyrosine kinase inhibition as a treatment strategy in AD.

## Conclusions

The current Phase Ib trial demonstrates that AZD0530 is generally safe and well tolerated in patients with AD, with oral dosing yielding excellent CNS drug concentration. Together with our preclinical data, the Phase Ib study results support the dosing regimen for a larger ongoing Phase IIa trial in AD of 100 to 125 mg AZD0530 daily (NCT02167256).

## Abbreviations

Aβ: amyloid-beta
Aβ_o_: oligomeric amyloid-beta
AD: Alzheimer’s disease
ADAS-cog: Alzheimer’s Disease Assessment Scale – cognitive subscale
ADCS-ADL: Alzheimer’s Disease Cooperative Study – Activities of Daily Living Inventory
CDR-SOB: Clinical Dementia Rating Scale – Sum of Boxes
CMRgl: cerebral metabolic rate for glucose
CNS: central nervous system
CSF: cerebrospinal fluid
FDG: fluorodeoxyglucose
IC50: half maximal inhibitory concentration
MMSE: Mini-Mental State Examination
NPI: Neuropsychiatric Inventory
PET: positron emission tomography
PrP^C^: cellular prion protein
SAE: serious adverse event
sCTX: serum C-telopeptide of type 1 collagen
sROI: statistical region of interest
ULN: upper limit of normal
